# Isolation in the COVID-19 pandemic as re-traumatization of war experiences

**DOI:** 10.3325/cmj.2020.61.371

**Published:** 2020-08

**Authors:** Igor Okorn, Sabina Jahović, Maja Dobranić-Posavec, Jovana Mladenović, Anton Glasnović

**Affiliations:** 1 Psychoanalytic Peer Group “Sophia,” Ljubljana, Slovenia; 2 Psychoanalytic Peer Group “Sophia,” Belgrade, Serbia; 3 Psychoanalytic Peer Group “Sophia,” Sarajevo, Bosnia and Herzegovina; 4 Psychoanalytic Peer Group “Sophia,” Zagreb, Croatia; 5 *Croatian Medical Journal*, Zagreb, Croatia

“What must happen is not a misfortune. Misfortune is only that what should not happen, but still happens. Misfortune is always caused by people. Nature rules with that what must happen.” (Borislav Pekić, Rabies)

We live in strange and dangerous times. The COVID-19 pandemic and restrictions imposed to contain it have in many ways adversely affected our daily functioning, with self-isolation and lockdown having a particularly damaging effect on mental health. This situation has caused different physical, psychological, and social manifestations on an individual, group, and social level. Individuals are experiencing anxiety, fear, panic, or paranoia, while communities and society are experiencing a crisis, with authorities desperately searching for appropriate responses. Both individuals and society as a whole are facing trauma.

This pandemic is often referred to as a “war-like situation.” The terms “war” and “pandemic” are used almost interchangeably to describe the current situation, but are these terms equivalent? Arguably, both war and pandemic lead to the collapse of the external safe environment and the phenomenon of “pathology of boundaries.”

The historical perspective of wars around the world, including those in the former Yugoslavia, confronts us with the experience of violated borders, breakdowns, destruction, loss of authority, disintegration, devastation, and a lack of empathy and humanity. In a war environment, both the individual and the society are faced with a different way of functioning, the need for adaptation, which undoubtedly generates painful, traumatic experiences.

In this article we present our clinical work with several patients from the former Yugoslavia in whom the pandemic caused a re-traumatization of the war experience. The first aim is to show the similarities and differences between wars and pandemics in terms of the way we are facing the situation. Traumatic experiences of pandemic and war are different in “their nature” and similar and different in their “pathological effects and consequences.” The second aim is to show that psychoanalysts can offer safe enough space and time, and an-other person, for re-traumatized patients to repair individual and collective trauma. The third aim is to show that individual trauma can only be repaired through both the individual psychotherapeutic process and the group thinking and dreaming processes. The individual trauma can only be elaborated, worked-through, and repaired on a collective level.

The purpose of this article is not sociological consideration, or a political analysis. As psychoanalysts, we would like to offer one interpretation of the connection between the pandemic and war experiences, through an analysis of clinical material from individual practice, which was also discussed in the peer group processes. In this way, we will try to present how we can start transforming trauma and re-traumatization into a process of reparation.

## Peer group and its functioning

The psychoanalytic peer group “Sophia” is a group of five psychoanalysts from four countries of the former Yugoslavia. The clinical material presented here is the material from our clinical practice. The material and processes from the individual psychoanalytic work of our members were brought to the group and were re-thought and re-dreamed in the group processes. Individual clinical work of re-traumatized patients was elaborated again on a collective level. Our members from Sarajevo and Belgrade used the group processes and “group mentality” as a safe-enough environment with functional barriers. In this way, the analyst could broadly and safely work through her own clinical material and later bring “the collective, group material” into the analytic setting. The patients of our two group members “became” patients on a collective level (of our group) and were “involved” in parallel thinking and dreaming processes (in the individual setting and on the group level).

Wilfred Bion says that when meeting an-other person, an individual “loses his own mind and identity.” Two people are already a group. Let us add that in addition to losing one’s mind, one is getting a “new individual and collective, group mind and identity” – “group mentality,” which is in constant process of changing. He calls this the proto-mental system of the group. The processes in the group function on the level of “basic assumptions” when the group is faced with primitive ways of connecting and disconnecting. In crisis situations, such as war or pandemic, very extreme and pathological processes in the group prevail. These are paranoid and schizoid states of mind (of collective/group mentality), when destruction, hatred, killing, colonizing, or torturing an-other group are viewed as “normal.” We deal with a group that is losing its mind – a group (or society) that goes insane. The chaos reigns and the group in despair tries to find a narcissistic leader – the magical savior-dictator and ideology that will save them all and forever. This solution is only temporary and decreases the overwhelming anxiety, fear, and paranoia, and will soon end in another collapse of the system, another broken and dysfunctional barrier, another war, and another collective trauma (1,2). How can we prevent this from happening again during the pandemic and in the future? Is “reparation” instead of repetition possible? And how?

The analysts working with patients who have experienced collective trauma and those who assimilated it trans-generationally need to expand their mental container (the capacity for thinking about trauma) (3). The analyst faces a difficult task that threatens his or her container and symbolic function, which can transform the “unconscious” into a “participating” witness, an experience after which neither of them can be the same. This is where the peer group comes into play as an “extended container” for the analyst, capable of metabolizing non-metabolized contents, thus preserving the symbolic function of the analyst. This experience results in a common psychic reality, an analytical intersubjective third (4), which belongs both to the analyst and the patient, making both mental containers “bigger” (5). In this space, the patient is not passively subordinated to the analyst, and there is place not only for personal developmental dynamics related to the parental care or neglect, but for other dimensions of human relationships. We allow ourselves to be touched by someone's pain in the space and time where we share information, energy, hope, and common knowledge (6).

## The most important theoretical considerations regarding trauma and re-traumatization

When faced with a danger, people who have not had the privilege of living in “war-free zones” for a few generations immediately start to link that particular danger to war. This can be attributed to an individual, collective, and trans-generational traumatization and re-traumatization. Here, psychoanalysis has a lot to offer, as it deals with various types of traumas and unconscious links to a broad array of symptoms.

These points were first addressed by Sigismund Freud, who was dealing with the concept of trauma all his life. At the very beginning (7) he equated the notion of trauma with infantile sexual trauma, which he later somewhat modified by recognizing the inner reality and phantasmatic life (8). Freud connected war neuroses that emerged during and after the First World War with anxiety, and replaced the term war neuroses with the term traumatic situation (9). Traumatic situation resulted from the interplay of inner and outer reality, which activates the entire universal and individual phantasmatic arsenal. In a traumatic situation, the emphasis is on the experience of loss – which puts the subject in a state of complete helplessness, motor and mental impotence amid a repeated eruption of internal and external stimuli. Indirectly, the subject, in an unsuccessful attempt to master trauma, connects the death instinct, traumatic situation, and repetition of compulsion. Nothing of the individual’s trauma can be repeated or remembered. In modern psychoanalysis, trauma refers to a situation that involves not only the subject and the breaking of his or her stimulus barrier, but also the sense of helplessness and the world of interpersonal relations and “object connections,” two terms that are not equivalent (8).

Subsequently, Michael Balint was the first to develop the concept of situational trauma, placing the object in external reality, thus putting the mother-child relationship, ie, their harmony, in the foreground (10). In contrast, Melanie Klein equated trauma with anxiety and the subject's ability to master it throughout his or her life (11). Masud Khan further defined the term “cumulative trauma” (12) and identified the mother as a shield and auxiliary ego. The place of trauma is the place of shield rupture, which occurs silently and invisibly during the developmental process and can only be understood retrospectively.

The theoretical concepts were expanded by Leo Rangel (1967), who introduced “vulnerability to trauma,” a very important clinical indicator that distinguishes traumatic from pathogenic (13). According to this notion, there is no trauma without an object, regardless of whether the object makes the pathogenic role of trauma clearer or if it blurs it. He says that regarding the nature and destiny of object relations (relation between the subject and object), we should maintain basic understanding of the economic theory of trauma in its pure form.

In an attempt to define collective trauma, Dori Laub emphasizes the demolition of empathic protective shield, made up of internalized primary objects, which makes the person lose confidence in good objects that have the capacity to give meaning to experience (14). What remains is “a hole of emptiness” in the place where something existed. Along the same lines, Marianne Leuzinger Bohleber (2010) emphasizes the damage done by inner and self-object representations in collective trauma (15). Basic trust in the world collapses, a trust that Jose Bleger describes as an agglutinative core that lies at the root of the psyche, which needs an environment to ensure de-symbiosis and individuation (16).

In line with modern theories thus presented, Rene Kaes and others stress that collective trauma damages the semipermeable membrane between reality and fantasy, and the subject is reduced to an anonymous individual (17-21). Social trauma is trans-individual, because besides intrusive violence, there is also an invasion of the common space. According to him, in addition to its unconscious foundations, the essential role of the psyche is the processing of differences, which can always trigger violence.

One of the most important tasks of analysts is to “tame” the trauma (very often, this metaphor is also used for the epidemic curve). The trauma can be tamed with the help of metaphors, with finding a name for traumatic experiences, in order to place it in a certain conceptual framework and thus limit its destructive power. Besides naming, we need to give meaning and explanation to experiences, which, according to Willie and Madeleine Baranger, is the place where psychoanalysis begins and where the analytical couple is born (8).

What we call an individual human being, a psyche, a container, an analytical setting, a group, a social group, an institution, a nation is not a closed system. It is a system that is in a constant dialectical relationship between outside and inside. Furthermore, the relation between inside and outside is characterized by a complex dynamism and manifestations of different psychological material, such as emotions, dreams, events, memories, sensations, associations, which give rise to turbulence, constant change of states, catastrophic points. The boundary or barrier between inside and outside is something that we may use when describing a precise line of separation or a buffer zone. When we deal with boundaries in human relations (when two people, two groups, or two nations, encounter/clash) and in psychoanalytical sense, we talk about their strength, elasticity, rigidity, permeability and capability of holding, containing a situation of and within an encounter/clash. When we talk about a damaged, broken, ruptured barrier and its functioning, we talk about the “pathology of boundaries” (22).

Damage of different quality and quantity may have different causes and different consequences. Paranoia is one of them. One of the extremes is a complete barrier destruction, which devastates the system and individual, namely causing chaos in a group or psychosis in an individual. The other extreme is when the barrier becomes hermetically sealed, non-permeable, with no elasticity. This extreme may cause a catatonic and autistic state of functioning, a complete isolation of the individual from the outside world.

## Excerpts from clinical material

During the pandemic, all our patients felt that something had changed. Moreover, all patients began to see the resemblance between the epidemic and war events. The same thing happened to psychoanalysts – we experienced a change in our feelings toward ourselves and our environment, especially patients. For example, the analyst from Sarajevo said that she “returned home unusually tired and exhausted” although her workload was comparable to that before the outbreak. She explained that she, and especially the patients, had a reduced processing capacity. One young male patient described his condition in the following way: “I feel lonely and can't work, and just lie in bed,” whereas a young female patient stated that she was “in panic because of her parents and son.” All these reactions of passivity and mortification could be a result of a traumatic experience during the siege of Sarajevo. Even so, some patients who more directly experienced war trauma also linked it directly to the epidemic. Quarantine isolation resembled the isolation experienced during the exile. The epidemic shook like an earthquake all those who were affected by the war and other traumas, and who established a fragile psycho-sociological balance within their environments, and forced them to regress. In reality, during the epidemic, an earthquake hit Zagreb and further “shook” the whole area and its fragile defenses after the war trauma. Consequently, one young female patient from Sarajevo said that she “panicked after the earthquake in Zagreb and felt helpless, which made her angry because of a reduced possibility of control.” One middle-aged female patient from Zagreb wondered “what awaits us next,” referring to the plagues of Egypt described in the Bible.

On the other hand, the “shaking of the sociological setting” sometimes caused quite opposite feelings. Another young female patient from Sarajevo stated that “the COVID epidemic put us all in the same situation” and that she “overcame her insecurities by cooking and nurturing flowers, which significantly relaxed her.” A young female patient from Belgrade described the lockdown experience “as her private space and time, freedom and security, and stability and change, in spite of the reality of going to work almost every day in a situation of danger.” This patient, although young, without memory of the war events, was still strongly marked by the war on an unconscious level. Her date of birth coincides with a major battle, a fact that stimulated certain internal processes and allowed her to start “moving” something inside herself. She stated that “some unconscious process happened and something has been opened.” She gained “the ability to explain how she feels, and the ability to feel love without needing to identify in total, and intimacy and sharing were possible again.” She found the time to think without guilt about things that are “stupid but dear to me,” such as going to the hairdresser’s or beautician’s. As if she was introduced into “something that gives life lightness and carless tone.” Before that, she started her analytic travel by talking about clothes for older women that her mother bought for her, as if she was 40 rather than 28. During the lockdown, she started wearing “sports clothes” and “casting aside the need to impress anyone.”

Therefore, the patients experienced opposite reactions to the pandemic, and it was up to analysts to detect the change of environment. However, all these reactions, prompt analysts’ and delayed patients’, were connected to war trauma, as if any new trauma “short-circuited” to war trauma.

## Discussion

Let us now discuss our patients' reactions provoked by the war and pandemic in relation to the “pathology of boundaries.” Two described extreme situations in war and pandemic are both traumatic but they differ in clinical presentation. Either in war or in a pandemic, there are different protective reactions and measures on an individual and collective level. In the presented clinical material, we can easily observe the “pathology of boundaries.” Both COVID-19 pandemic and war are traumatic for an individual, a group, or a system. Both situations are repetitive and experienced as (re)traumatization on the level of barriers. We can recognize dysfunctional or collapsing barriers in the individual and in the system.

In analytical space – our clinical setting – a safe-enough container, these patients can establish a relation with an-other person (the analyst). In this space, the patients in Sarajevo and the patient in Belgrade could experience their dysfunctional barrier and repair it, which prevented them from being re-traumatized by the pandemic situation.

According to theoretical ideas, our patients are trying to transform their trauma from a “purely economic” (which is connected to symptoms and not to the thinking processes) to a trauma that will be assimilated into the history of the individual and the group. Often, brutal events (accidents, massacres, war, genocide, or holocaust), if they remain meaningless, become only incidents and intruders destined for repetition. Thus, analytical work creates the opportunity to transform repetition and experience of death to thinking and historicization (23).

Furthermore, patients were sharing the ideas and memories of traumatic war experiences in Sarajevo under siege. The analyst helped them with her own counter-transference feelings and with dreaming with them about their experiences in a state of reverie. This process helped the patient and the analyst to deal with the material in a consulting room. The analyst, being aware of her own traumatic experiences, could offer them the space – the container with safe and functional barrier – where patients could evacuate and express their own unbearable feelings and emotions, and this would help them understand what was happening. Later, when lifting of the restrictions reestablished the boundary and function of the barrier between inside and outside, the level of unbearable individual and collective anxiety decreased.

The psychoanalyst assumes the role of a participating witness (24,25). The analyst, as a member of human community, can also be traumatized by the same event and can participate in the discourse, which re-actualizes the experience that allows the analytical couple to share knowledge and memories. They could use that knowledge and memories for identification processes, without feelings of persecution and tendencies to avoid (6).

It is interesting how “isolation” and the autistic position helped one of our patients to repair early traumatic experiences in her family. In an isolated position, this patient did not just take part in remembering and repeating, but repairing and being creative. She used the analytical frame and setting and her relation with the analyst to feel safe enough, which caused or triggered her memories that were dreamed, thought, and worked through in an isolated-autistic position. She at last became a creative adolescent, and in parallel re-experienced and worked through her traumatic situations with her mother. At the same time, the isolation brought up images and memories of her childhood related to her birthday that coincides with a major battle and a picture from war in her home.

## Conclusion

Let us finish with two possible metaphors that describe our profession. In both individual sport and team sport, a professional needs a team to progress and develop. In team sport, it is the individual's team or group, while in the individual sport it is a team of trainers and staff.

The same applies to artists or scientists. Artists cannot work on an individual level. Even if they isolate themselves in an autistic position, when presenting their work to the public they will be faced with a group (collective mind). The artist starts to communicate with “an-other people mind-group mentality.” Only when an artist is faced with a group and is “recognized in a group,” he or she gets the validation and confirmation of the artwork in a larger container – society.

The COVID-19 pandemic has shown us that we can be connected as human beings and express empathy, solidarity, and humanity. War, unfortunately, gives rise to quite the opposite experiences – human beings often lose their “human faces,” and groups and systems are destroyed or fight each other. As a society, we will probably soon find a cure for COVID-19. However, we will certainly need much more time to find the “cure” for the collective and individual (trans-generational) trauma caused by wars and their consequences. We hope that this text could give some directions toward reaching this goal.
